# Adrenal Insufficiency in Cystic Fibrosis: A Rare Phenomenon?

**DOI:** 10.1155/2018/3629031

**Published:** 2018-03-13

**Authors:** Sébastien Préville-Ratelle, Adèle Coriati, Aurélie Ménard, Isabelle Bourdeau, François Tremblay, Yves Berthiaume

**Affiliations:** ^1^Clinique de fibrose kystique, Centre hospitalier de l'Université de Montréal (CHUM), 1051 rue Sanguinet, Montréal, QC, Canada H2X 0C1; ^2^Institut de recherches cliniques de Montréal (IRCM), 110 Avenue des Pins, Montréal, QC, Canada H2W 1R7; ^3^Centre de recherche Centre hospitalier de l'Université de Montréal (CRCHUM), 900 rue Saint-Denis, Montréal, QC, Canada H2X 0A9; ^4^Département de médecine, Faculté de médecine, Université de Montréal, 2900 boul. Édouard-Montpetit, Montréal, QC, Canada H3T 1J4

## Abstract

**Background:**

The prevalence of adrenal insufficiency (AI) in cystic fibrosis (CF) is unknown. The frequent use of glucocorticoids (inhaled or systemic) may induce the long-term suppression of the hypothalamic-pituitary-adrenal axis.

**Methods:**

We reviewed the results of adrenocorticotropic hormone (ACTH) stimulation tests done over a 10-year period to evaluate adrenal function in 69 CF patients of the CHUM CF clinic. Clinical characteristics of AI patients were compared to adrenal-sufficient (AS) patients.

**Results:**

AI was confirmed in 33 of the 69 CF patients. A higher rate of dysglycemia (*P*=0.022) and of *Aspergillus* positive culture (*P*=0.006) was observed in AI patients compared to AS patients. Weight, CFTR genotype, and pulmonary function were comparable between AI and AS patients. The use of systemic corticosteroids (SC) prior to the diagnosis of AI was observed in 42.4% of patients. Compared to AI patients without SC, SC-treated AI patients were older and had a higher rate of allergic bronchopulmonary aspergillosis.

**Conclusion:**

This study is the first to systematically examine the presence of AI in the largest cohort of CF patients studied to date with a prevalence of 8%. Patients treated with corticosteroids and those colonized with *Aspergillus* have a greater risk of AI.

## 1. Introduction

Adrenal insufficiency (AI) is the result of decreased hormonal production from the cortex of the adrenal gland. AI may be caused by adrenal failure itself (primary AI) or by disease of the pituitary or hypothalamus (secondary or tertiary AI, resp.). The most common cause of secondary AI, however, results from the chronic therapeutic use of glucocorticoids which can suppress the hypothalamic-pituitary-adrenal axis (HPA) and lead to insufficient cortisol production in response to stress [1]. AI is associated with a potential risk of adrenal crisis and severe complications if the diagnosis is delayed. The short adrenocorticotropic hormone (ACTH) stimulation test (Cortrosyn 250 *μ*g) is the most commonly used dynamic evaluation to confirm the diagnosis of AI [2].

Although AI induced by inhaled corticosteroids (ICSs) has been documented in asthmatic adult populations [3], no studies have evaluated the prevalence of AI in the adult cystic fibrosis (CF) population. Trials on the use of ICSs in CF for the treatment of allergic bronchopulmonary aspergillosis did not systematically evaluate adverse events, although the total daily dose of ICSs was at the threshold of adrenal suppression in most studies [4]. To date, there have been only a few case reports of HPA axis suppression in patients with CF caused by the combination of ICSs (mainly fluticasone) and itraconazole, which inhibits cytochrome P450 consequently decreasing the metabolic clearance of ICSs [5–7].

Our clinical experience at the Centre hospitalier de l'Université de Montréal (CHUM) CF clinic suggests a nonnegligible prevalence of AI among our patients. Therefore, we reviewed the results of the ACTH stimulation test performed over a 10-year period to evaluate adrenal function in 69 CF patients and compared the clinical characteristics of CF patients with AI to those of adrenal-sufficient (AS) patients.

## 2. Methods

This is a cross-sectional retrospective study approved by the Research Ethics Committee and CHUM Institutional Review Committee. This work was carried out in accordance with the Declaration of Helsinki (1964). The records of 385 patients attending the CF clinic between January 1, 2007, and December 31, 2016, were reviewed. Cases were selected from the CF clinic database and from the computerized laboratory system at CHUM. Inclusion criteria for the study were (1) age 18 or more and (2) a documented ACTH stimulation test (Cortrosyn stimulation test).

Two different ACTH stimulation tests have been performed: the low dose (LDCT, Cortrosyn 1 *μ*g) and the high dose (HDST, Cortrosyn 250 *μ*g). The HDST and the LDCT are the alternative less-invasive methods used in hospitals and clinics to assess adrenal function in patients with suspected chronic AI. However, several meta-analysis studies have demonstrated that the LDCT is more sensitive than the HDST to diagnose secondary AI, such as in patients taking corticosteroids, since the HDST uses supraphysiologic doses of ACTH [8, 9]. For these reasons, the LDCT is now preferred. The LDCT must be done by people well-informed in the preparation and the administration of the 1 *μ*g Cortrosyn [10]. To ensure appropriate testing at our CHUM clinic, the hospital pharmacist is responsible for preparing the Cortrosyn syringe and the nurse uses this preparation to perform the test. In both tests, plasma cortisol levels were measured at baseline as well as 30 and 60 minutes after intravenous injection. Normal adrenal function was defined as a minimum plasma cortisol concentration of 500–550 nmol/L before or after ACTH injection in both low-dose and high-dose tests. When peak cortisol was below this threshold, the diagnosis of AI was confirmed and treatment with hydrocortisone (Cortef) was started. Patients with CF who underwent an organ transplantation were excluded from the study. Of the 69 CF patients that underwent an ACTH functional test, 36 were categorized as AS, whereas 33 were diagnosed with AI. Amongst the 36 patients with AS, 33 underwent the LDCT, 2 did the HDST, and 1 patient did an insulin tolerance test [11]. Between the 33 patients with AI, 28 did the LDCT, 2 underwent the HDST, and 3 were diagnosed with AI because they had undetectable (significantly low concentrations in the laboratory results) plasma cortisol levels. Physicians of the CF clinics will prescribe the ACTH test if the patient has a low blood cortisol concentration in the morning. This screening is done once a year with the routine biochemistry monitoring of the patient's condition. An ACTH test is also prescribed if the patient is experiencing significant fatigue that is unrelated to his clinical status. In other words, the patient feels always tired and does not have a lot of energy, in presence of stable lung function and weight and in absence of significant systemic inflammation.

Demographics (age, sex, weight, and body mass index (BMI)), pulmonary disease characteristics (forced expiratory volume in 1 sec (FEV1), forced vital capacity (FVC), residual volume (RV), oxygen dependence, CFTR genotype, and noninvasive ventilation support), comorbidities (pancreatic and hepatic dysfunction as evidenced by abnormal level of hepatic enzymes or presence of cirrhosis), dysglycemia (diabetes or impaired glucose tolerance), allergic bronchopulmonary aspergillosis (ABPA), microbiological data (bacteria, fungi, and non-TB mycobacteria), biochemical and hematological data (median value over the past year of hemoglobin and white blood cells, and fasting serum cortisol), and results of the functional ACTH test were collected from all of the patients. The total number of intravenous antibiotic treatments and hospitalizations during the two years preceding the diagnosis of AI as well as the use of ICSs, systemic corticosteroids, and chronic antibiotic therapy (inhaled) were also documented.

Values are expressed as median and interval range for each study group (AS versus AI, and AI with or without systemic corticosteroid). Statistical significance of the differences between study groups was determined by Mann-Whitney Student's *t*-test, as well as by chi-square (chi^2^) regression analysis for genotype and hepatic function. All statistical analyses were done with SPSS for Windows software (version 17.0, SPSS Inc., Chicago, IL, USA). A probability value of *P* ≤ 0.05 was considered statistically significant.

## 3. Results

Over the 10-year period, 385 patients diagnosed with CF were seen at the CHUM CF clinic. In this group of CF patients, 69 patients underwent functional tests, of which 36 patients (41.7% men) had normal responses (AS) and 33 patients (48.5% men) had a confirmed diagnosis of AI. From these data, the prevalence of AI in this cohort was calculated to be 8.6%. Their clinical characteristics are detailed in Tables 1 and 2. The median age and BMI of CF patients with AI were comparable to those that had AS (28.0 years and 21.6 kg/m^2^ versus 30 years and 20.9 kg/m^2^, resp.) (Table 1). The most frequent genotype was the homozygous delta F508 mutation in both AS and AI (58.3% and 60.6%, resp.). Interestingly, the proportion of patients with dysglycemia was statistically higher in AI patients compared to AS patients (87.9% versus 63.9%, *P*=0.022). Pancreatic insufficiency, hepatic dysfunction, and presence of ABPA were not increased in AI patients. No difference was observed when comparing parameters of the pulmonary function test (FEV1, FVC, and RV) as well as WBC values between both adrenal function groups (Table 2). Patients with AI were more likely to take ICSs and less prone to use chronic inhaled antibiotics (*P*=0.005, Table 2). However, the number of hospitalization and the number of IV antibiotic treatments 2 years prior to diagnosis were comparable between AS and AI patients. Finally, the sole microorganisms that had a significant difference of proportionality between AS (17.1%) and AI (48.5%) patients were *Aspergillus* and fungi (*P*=0.006, Table 2).

Systemic corticosteroid therapy prior to the diagnosis of AI was observed in 14 patients with a median duration of administration of 18 months (varying between 1 week and 74 months) (Table 2). Of these 14 patients, all but one received corticosteroids for more than a month in the year prior to the diagnosis. When comparing with AI patients who did not receive systemic corticosteroids, AI patients taking systemic corticosteroids were older (36.0 years versus 26.0 years, *P*=0.019) (Table 3). Interestingly, the use of systemic corticosteroids was justified by the diagnosis of allergic bronchopulmonary aspergillosis (ABPA) in 9 cases (64.3%). In addition, AI patients with systemic corticosteroid had significantly lower levels of cortisol at 30 minutes during the ACTH stimulation test (*P*=0.047). Treatment with systemic corticosteroids was not associated with antifungal treatment. The median number of hospitalizations and of intravenous antibiotic treatments in the two years preceding the diagnosis and the profile of bacterial and fungal infection and medication are detailed in Table 4. ICSs were used in 92.9% of AI patients with systemic corticosteroids. In the 19 patients who had no recent exposure (<2 years) to systemic corticosteroids, all but one were using ICSs. The ICSs used were fluticasone or budesonide mainly at high dosage and sometimes in combination with ciclesonide. The detailed clinical findings of these patients are reported in Tables 3 and 4.

The chronic use of opiates was the suspected etiology of AI in 2 cases. The prolonged intake of voriconazole for the treatment of *Scedosporium apiospermum* was observed in 2 patients. Use of other antifungal agents in the Montreal CF cohort has not been identified (data not shown).

## 4. Discussion

AI is the inability to produce sufficient amount of steroid hormones (primarily cortisol) from the adrenal glands. Until now, only individual case reports relating CF and AI have appeared in the literature. This is the first study that characterizes a large group of adult patients with CF that had developed AI. Our observations show that, compared to the AS subgroup, the AI subgroup of CF patients exhibited a higher frequency of dysglycemia and had a higher percentage of patients colonized with *Aspergillus*/fungi. AI patients on systemic corticosteroids were also older. Finally, the utilization of ICSs or systemic corticosteroids was more frequent in patients with AI. More interestingly, more than half the AI patients on systemic corticosteroids had ABPA compared to none in the subgroup of patients not taking systemic corticosteroids.

We are the first to estimate the prevalence of AI in an adult CF population. Our data, obtained following review of the clinical records of over 385 CF patients, suggest that the prevalence of AI in our CF population is between 8% and 9%. This value may be underestimated due to the difficulty of recognizing AI and the lack of systematic screening. Although significant, this prevalence is much lower than the one that has been observed in non-CF bronchiectasis. Indeed, in a study of 40 consecutive patients with non-CF bronchiectasis, Rajagopala et al. [12] reported that 40% of patients had a reduced response to functional testing. No association with inhaled or oral corticosteroids therapy, or severity of impairment was identified in this study. In a British study of non-CF bronchiectasis, adrenal suppression was found in 23.5% of subjects not receiving ICSs and in 48.5% of patients receiving it [13]. Skov et al. observed AI in 11 out of 25 patients with CF treated both with itraconazole for ABPA and budesonide [14], while one of the 11 patients treated with itraconazole alone (without budesonide) had AI. More recently, Gilchrist et al. found a higher prevalence of HPA axis suppression in 10 out of 12 patients with CF receiving both itraconazole and inhaled fluticasone compared to patients with CF receiving inhaled fluticasone alone [15]. In all these studies, there was a selection of patients based on either the therapy administered or their clinical status. This might explain the difference in the prevalence observed. A prospective study will be necessary to determine more precisely the prevalence of this condition in CF.

Dysglycemia and *Aspergillus*/fungi colonization also appear as potential risk factors for AI. Our study showed an increased prevalence of dysglycemia (87.9%) in AI compared to AS patients (63.9%). Although this association could be secondary to the chronic inflammatory response associated with diabetes, it could also be a confounding factor since it is well known that chronic use of corticosteroids can lead to heightened blood glucose levels and diabetes [16]. Chronic inflammatory response associated with CF could also explain the presence of AI in patients who did not take corticosteroids. Indeed, Mastorakos et al. have demonstrated that prolonged inflammatory stress induced by a sustained high level of interleukin- (IL-) 6 decreases the reactivity of the HPA axis over time [17]. Inadequate cortisol production has also been demonstrated in rheumatologic patients. The study by Straub et al. showed that levels of ACTH and cortisol, when related to IL-6 and TNF, were relatively lower in untreated patients with early rheumatoid arthritis and reactive arthritis as compared to healthy subjects [18]. Noni et al. reported an association between *Aspergillus* colonization and duration of ICS treatment [19]. A future case-control study is needed to validate these findings, to more precisely determine the real prevalence of AI and its risk factors, and to evaluate the clinical course and repercussions of AI on the quality of life of CF patients. Our observations suggest that CF patients who have an abnormal glucose status (glucose intolerant or CF-related diabetes) or are colonized with *Aspergillus*/fungi or have received systemic corticosteroids as well as have ABPA might benefit from a systematic screening of adrenal function.

Clearly, the utilization of corticosteroids plays a major role in the suppression of the HPA axis in CF patients. In our study, the majority of patients with AI were treated with ICSs for several years. Although the therapeutic use of ICSs remains controversial in the management of CF [4, 20], it remains widespread. There are multiple factors that might explain the association between ICS use and AI in CF. First, chronic airway inflammation is associated with pathological angiogenesis in the airway [21, 22]. It is suggested that this increase in vascularization in the airway and lungs might lead to increased absorption of corticosteroids into the systemic circulation. Second, since a significant quantity of inhaled corticosteroid is swallowed into the digestive tract, the use of high doses will lead to increased levels in the systemic circulation [23]. Besides absorption, other factors play an important role in the suppression of the HPA axis by ICSs. Indeed, the bioavailability of inhaled compounds is not uniform [24]. Therefore, the ICS compound that has the lowest oral bioavailability would be the best option to decrease undesired corticosteroid effects [24, 25]. Other factors that can affect corticosteroid availability include systemic clearance, receptor affinity, and protein binding capacity [24]. Finally, plasma cortisol concentration varies significantly during the day, with the highest levels measured early in the morning at around 8 AM and the lowest at midnight [24]. Thus, to minimize the secondary effects of corticosteroids on cortisol suppression, it becomes important to consider the elimination half-life of the inhaled compound as well as the drug administration time so that the peak drug level is in phase with the natural cycle of endogenous cortisol. Additional studies are essential to study the pharmacodynamics of ICSs specifically in CF.

The ACTH stimulation test requires multiple steps for its preparation, and it has to be done by experienced personal and is time consuming. Identifying and proposing a management plan where ACTH stimulation is not the primary test that is done but it should be used only to confirm the diagnosis before beginning therapy would be important [26]. A potential management plan including an easy screening tool that can be done during a routine clinical visit will need to be evaluated in future studies.

## 5. Conclusion

This study is the first to systematically examine adrenal function in a cohort of CF patients. Our analysis would suggest that the prevalence of AI is at least 8% in our adult CF clinic. Therapeutic use of inhaled and systemic corticosteroids was often identified in patients with adrenal suppression. Considering the lack of clear clinical benefit of ICS therapy in CF, our results show the need for additional studies to reduce both the burden of medication and the potential adverse effects of corticosteroid therapy in CF patients.

## Figures and Tables

**Table 1:** Clinical characteristics of patients with CF according to their adrenal function (*N*=69).

	Sufficient	Insufficient	*P* value
*Demographic*			
Number of patients	36	33	
Sex (% men)	41.7	48.5	0.572
Age (years)	30.0 (19.0–67.0)	28.0 (19.0–55.0)	0.648
Weight (kg)	54.1 (42.3–83.9)	61.0 (41.1–79.1)	0.084
BMI (kg/m^2^)	20.9 (17.5–28.6)	21.6 (17.1–27.5)	0.079
Genotype (% yes)			
Homozygote delF508	58.3	60.6	
Heterozygote delF508	38.9	30.3	0.458^∗^
Others	2.8	9.1	
*Comorbidities*			
Glucose tolerance (% yes)			
Normal	36.1	12.1	0.022
Dysglycemic	63.9	87.9	0.022
Pancreatic insufficiency (% yes)	91.7	87.9	0.605
Hepatic dysfunction (% yes)	30.3	11.1	0.041
Home oxygen therapy (% yes)	5.6	15.2	0.190
Noninvasive ventilation (% yes)	2.8	6.1	0.507
ABPA (% yes)	16.7	27.3	0.290

BMI, body mass index; dysglycemic is presence of either glucose intolerance or diabetes; hepatic dysfunction is either abnormal level of hepatic enzymes or presence of cirrhosis; ABPA, allergic bronchopulmonary aspergillosis. Median and interval range are shown. *P* value ≤ 0.05 represents Mann-Whitney Student's *t*-test. ^∗^Chi^2^ regression analysis.

**Table 2:** Paraclinical characteristics of patients with CF according to their adrenal function (*N*=69).

	Sufficient	Insufficient	*P* value
*Pulmonary function test*			
FEV1 (% pred)	44.6 (22.0–120.0)	52.9 (27.5–82.6)	0.509
FVC (% pred)	69.9 (32.8–113.8)	72.6 (51.0–97.8)	0.885
RV (% pred)	177.0 (80.0–414.0)	197.5 (126.0–406.0)	0.271
*Biochemistry*			
Fasting cortisol (nmol/L)	421.0 (191.0–954.0)	146.0 (12.0–335.0)	≤0.001
Cortisol (nmol/L)			
1 *μ*g functional cortrosyn test (*n*)	33	28	
0 minutes	388.0 (113.0–852.0)	139.5 (10.0–382.0)	≤0.001
30 minutes	628.0 (502.0–1079.0)	354.0 (25.0–491.0)	≤0.001
60 minutes	510.0 (210.0–902.0)	300.5 (23.0–457.0)	≤0.001
250 *μ*g functional cortrosyn test (*n*)	2	2	
0 minutes	426.5 (407.0–446.0)	158.5 (26.0–291.0)	NA
30 minutes	637.5 (606.0–669.0)	325.5 (179.0–472.0)	NA
60 minutes	678.0 (664.0–692.0)	309.5 (238.0–381.0)	NA
*Hematology*			
WBC (10^∗^9/L)	7.6 (4.1–10.8)	8.0 (2.55–17.5)	0.294
*Medication*			
Systemic corticosteroids (% yes)	0	42.4	≤0.001
Duration (months)	—	18.0 (0.25–74.0)	—
Inhaled corticosteroids (% yes)	66.7	93.9	0.005
Chronic inhaled antibiotic (% yes)	100.0	78.8	0.005
*Medical history*			
Number of hospitalizations (2 years prior to diagnosis)	2.0 (0–8.0)	2.0 (0–11.0)	0.593
Number of intravenous antibiotic treatments (2 years prior to diagnosis)	3.0 (0–8.0)	3.0 (0–11.0)	0.565
*Colonization/microbiology*			
Non-TB mycobacteria (% yes)	8.6	3.0	0.335
*Staphylococcus aureus* (% yes)	31.4	39.4	0.495
*Pseudomonas aeruginosa* (% yes)	77.1	81.8	0.636
*Aspergillus* and fungi (% yes)	17.1	48.5	0.006
*Stenotrophomonas maltophilia* (% yes)	25.0	30.3	0.625
Others (% yes)	19.4	12.1	0.410

FEV1, forced expiratory volume-one second; FVC, forced vital capacity; RV, residual volume; WBC, white blood cell count; TB, tuberculous; NA, not applicable. Median and interval range are shown. *P* value < 0.05 represents Mann-Whitney Student's *t*-test.

**Table 3:** Clinical characteristics of adrenal insufficient patients with CF with and without systemic corticosteroids (*N*=33).

Systemic corticosteroids	Yes	No	*P* value
*Demographic*			
Number of patients	14	19	
Sex (% men)	35.7	57.9	0.215
Age (years)	36.0 (21.0–55.0)	26.0 (19.0–39.0)	0.019
Weight (kg)	58.8 (50.0–79.1)	61.2 (41.1–72.8)	0.913
BMI (kg/m^2^)	21.3 (19.1–27.4)	21.7 (17.1–27.5)	0.971
Genotype (% yes)			
Homozygote delF508	57.1	63.2	
Heterozygote delF508	35.7	26.3	0.825^∗^
Others	7.1	10.5	
*Comorbidities*			
Glucose tolerance (% yes)			
Normal	7.1	15.8	0.459
Dysglycemic	92.9	84.2	0.459
Pancreatic insufficiency (% yes)	71.4	100.0	0.014
Hepatic dysfunction (% yes)	21.4	36.8	0.208
Home oxygen therapy (% yes)	7.1	21.1	0.278
Noninvasive ventilation (% yes)	0	10.5	0.217
ABPA (% yes)	64.3	0	≤0.001

BMI, body mass index; dysglycemic is presence of either glucose intolerance or diabetes; hepatic dysfunction is either abnormal level of hepatic enzymes or presence of cirrhosis; ABPA, allergic bronchopulmonary aspergillosis. Median and interval range are shown. *P* value < 0.05 represents Mann-Whitney Student's *t*-test. ^∗^Chi^2^ regression analysis.

**Table 4:** Paraclinical characteristics of adrenal insufficient patients with CF with and without systemic corticosteroids (*N*=33).

Systemic corticosteroids	Yes	No	*P* value
*Pulmonary function test*			
FEV1 (% pred)	56.9 (28.6–73.7)	44.1 (27.5–82.6)	0.536
FVC (% pred)	75.3 (51.0–96.4)	68.6 (56.4–97.8)	0.466
RV (% pred)	188.5 (128.0–221.0)	238.0 (126.0–406.0)	0.051
*Biochemistry*			
Fasting cortisol (nmol/L)	134.0 (25.0–254.0)	169.5 (12.0–335.0)	0.323
Cortisol (nmol/L)			
1 *μ*g functional cortrosyn test (*n*)	10	18	
0 minutes	124.5 (25.0–219.0)	167.0 (10.0–382.0)	0.415
30 minutes	314.0 (86.0–425.0)	398.5 (25.0–491.0)	0.179
60 minutes	279.0 (64.0–457.0)	315.5.0 (23.0–425.0)	0.388
250 *μ*g functional cortrosyn test (*n*)	1	1	
0 minutes	26.0	291.0	NA
30 minutes	179.0	472.0	NA
60 minutes	238.0	381.0	NA
*Hematology*			
WBC (10^∗^9/L)	9.4 (4.9–17.5)	7.6 (2.6–15.0)	0.074
*Medication*			
Inhaled corticosteroids (% yes)	92.9	94.7	0.823
Inhaled chronic antibiotic therapy (% yes)	78.6	78.9	0.979
*Medical history*			
Number of hospitalizations (2 years prior to diagnosis)	2.0 (0–6.0)	4.0 (0–11)	0.151
Number of intravenous antibiotic treatments (2 years prior to diagnosis)	2.5 (0–7.0)	4.0 (0–11)	0.212
*Colonization/microbiology*			
Non-TB mycobacteria (% yes)	7.1	0	0.244
*Staphylococcus aureus* (% yes)	42.9	36.8	0.731
*Pseudomonas aeruginosa* (% yes)	71.4	89.5	0.191
*Aspergillus* and fungi (% yes)	50.0	47.4	0.883
*Stenotrophomonas maltophilia* (% yes)	28.6	31.6	0.826
Others (% yes)	14.3	10.5	0.747

FEV1, forced expiratory volume-one second; FVC, forced vital capacity; RV, residual volume; WBC, white blood cell count; TB, tuberculous; NA, not applicable. Median and interval range are shown. *P* value < 0.05 represents Mann-Whitney Student's *t*-test.
